# Branching out: Resolving plant evolution through phylogenetic networks

**DOI:** 10.1002/aps3.70065

**Published:** 2026-06-18

**Authors:** George P. Tiley, Claudia Solís‐Lemus

**Affiliations:** ^1^ Department of Plant and Microbial Biology North Carolina State University Raleigh North Carolina USA; ^2^ Wisconsin Institute for Discovery University of Wisconsin–Madison Madison Wisconsin USA

The diversity of life is shaped by the interplay of multiple evolutionary processes. Some of these processes, like speciation, are well represented by a bifurcating tree. Others, like gene flow, hybridization, and introgression, are more appropriately described by networks. In plants, there are other common processes, such as polyploidy, reproductive mechanisms, and rapid shifts in life history characters, that can blur lineage boundaries and complicate evolutionary histories. As high‐quality genomic data have become increasingly accessible, it has become evident that some of the most persistent challenges in resolving relationships in the plant tree of life do not arise from insufficient data, but from the need to model these reticulate processes explicitly.

To better detect patterns of reticulate evolution in plants and infer the mechanisms underlying those patterns, new method development and increased accessibility of bioinformatic tools that especially consider plant biology are needed. However, methodological progress has not kept pace with the increasing amounts of genomic data and its now routine application to basic research in plant evolutionary biology. Inferring phylogenetic networks remains considerably more difficult than estimating trees, both statistically and computationally. Models that accommodate reticulation must also account for other sources of discordance, such as incomplete lineage sorting, leading to a rapid increase in complexity even for modest datasets. Moreover, the evolution of a hybridizing complex or even a single species can involve multiple overlapping events, producing network structures that are challenging to estimate and interpret. Practical limitations, such as computational scalability to large numbers of taxa, sensitivity to noise and missing data, and limited incorporation of biologically relevant processes, further constrain current approaches. As a result, there remains a substantial methodological gap to detect the signatures of reticulate evolution in modern plant phylogenomic datasets.

This special issue presents a collection of papers that showcase the breadth of current work on phylogenetic networks and related approaches for studying reticulate evolution in plants. The contributions include new tools for network visualization (Schliep et al., 2026), streamlined hybrid enrichment (Hyb‐Seq) phylogenomic workflows (Liu et al., 2026), haplotype assembly from long‐read amplicon data (Fakoya et al., 2026), detection of introgression from site‐frequency patterns with machine learning (McKenzie and Eaton, 2026), and statistical detection of ghost lineage introgression (Forsythe et al., 2026). These methodological advances are complemented by empirical studies that creatively harness the information within genomic data: repurposing population genomic datasets to characterize plant‐associated microbiomes (Gonzalez‐Ramirez et al., 2026), dissecting ancient hybridization and gene tree discordance across Asteraceae (Ellestad et al., 2026), and developing Hyb‐Seq‐based DNA barcodes for timber identification from wood specimens (Bellot et al., 2026), a historically challenging task yet critical to conservation biology.

Several contributions to this issue develop new methods to detect and characterize reticulate evolutionary history at different scales and levels of resolution. At the sequence level, Fakoya et al. (2026) introduce HapAsmbl, which reconstructs diploid haplotypes from multi‐locus Nanopore amplicon data, enabling fine‐grained studies of allelic diversity in polymorphic populations such as perennial ryegrass. Scaling up to broader phylogenetic comparisons, McKenzie and Eaton (2026) show how coalescent simulations with their program simcat can be used to train a neural network model to identify introgression signals from SNP frequency patterns across many quartet combinations simultaneously, extending the reach of invariant‐based inference well beyond the traditional quartet analyses. Yet, even a well‐supported introgression signal may be erroneous if its true source is an unsampled or extinct lineage. This challenge of missing lineages is addressed by Forsythe et al. (2026), who leverage branch length information to distinguish ghost lineage introgression from gene flow among sampled lineages in four‐taxon tests. The new Python package ghostbuster (Forsythe et al., 2026) should provide a reproducible approach to detecting ghost introgression, as demonstrated through reanalysis of previously identified introgression events in Brassicaceae.

In addition to analytical advances, the accurate representation of reticulate evolution depends on the infrastructure available to generate and communicate the underlying data. Liu et al. (2026) introduce HybSuite, which consolidates the major steps of hybrid‐capture phylogenomics from raw read retrieval and preprocessing to assembly, paralog handling, and species tree inference into a single reproducible Bash‐based workflow, lowering the practical barriers between sequencing data and biological interpretation. Once high‐quality datasets are in hand and analytical methods have been run, communicating the resulting complex evolutionary relationships requires equally capable visualization tools. Schliep et al. (2026) meet this need with tanggle, an R package extending the ggtree (Yu et al., 2017) and ggplot2 (Wickham, 2016) frameworks to support flexible, publication‐quality visualization of phylogenetic networks, including the integration of morphological and geographic data. Together, these contributions span the full arc from data generation to evolutionary inference, reflecting a field that is evolving both in its analytical modeling as well as the practical bioinformatics tools that are needed to reconstruct reticulate processes in the plant tree of life.

Beyond methodological development, the remaining contributions illustrate how phylogenomic approaches can reveal complex evolutionary and ecological patterns across diverse plant systems. Gonzalez‐Ramirez et al. (2026) draw attention to an often overlooked feature of genomic datasets: the off‐target reads that accumulate alongside intended sequence data. Using whole‐genome sequencing from the liverwort *Calasterella californica* (Hampe ex Austin) D.G. Long and T.X. Zheng, they characterize associated bacterial communities and show that these vary geographically across California, with *Methylobacterium* and related taxa dominating and spatial patterns likely tied to precipitation and temperature seasonality. The study makes a broader point as well: population genomic datasets can be repurposed to investigate host‐associated microbiomes, opening new windows into ecological interactions that would otherwise require separate sampling efforts.

Ellestad et al. (2026) confront a persistent challenge in plant systematics: pervasive phylogenetic discordance in Asteraceae, one of the most species‐rich families of flowering plants. Integrating Hyb‐Seq and transcriptomic data, they disentangle the contributions of hybridization, incomplete lineage sorting, and paralogy from ancient whole‐genome duplication to gene tree discordance. The authors found evidence of ancient reticulation particularly among South American lineages alongside widespread but difficult‐to‐interpret signatures of whole‐genome duplication. Recognizing that such analyses can be difficult to replicate, the authors pair their empirical results with a detailed tutorial designed to promote transparency and reproducibility in studies of complex evolutionary histories.

The question of how phylogenomic resources translate into real‐world utility finds a concrete answer in the final contribution. Bellot et al. (2026) turn to herbarium specimens in the mahogany family (Meliaceae) to develop a workflow for identifying short, informative DNA barcodes suitable for timber identification. By screening hundreds of nuclear and plastid loci from Hyb‐Seq and genome skimming data, they identify candidate regions that can be reliably amplified and sequenced from wood samples, achieving high rates of species‐level identification. The result is both a practical tool for tracking and regulating the timber trade and a demonstration of how legacy herbarium collections, combined with modern genomic approaches, can be mobilized to address urgent conservation challenges.

The contributions in this issue show that reticulate evolution research is impactful and can help bring clarity to complex plant evolution. Continuing to build methodological infrastructure in this area is an ongoing goal, as much remains to be resolved. More work on the statistical modeling of overlapping evolutionary processes and the computational scalability of network‐based inference with genomic data is needed, but the trajectory is clear: the study of reticulate evolution in plants is becoming at once more rigorous, more accessible, and more consequential, and the papers collected here reflect that momentum.

## AUTHOR CONTRIBUTIONS

C.S.‐L. wrote the first draft of the introduction, and G.P.T. and C.S.‐L. edited the introduction and approved the final version. Both reviewed initial proposals and handled manuscripts contributed to this special issue.
